# Opioid Use Associated With Higher Costs Among Patients With Inflammatory Bowel Disease

**DOI:** 10.1093/crocol/otab021

**Published:** 2021-04-14

**Authors:** Eva M Szigethy, Sean M Murphy, Orna G Ehrlich, Caren A Heller, Nicole M Engel-Nitz, Perry Meadows, John I Allen

**Affiliations:** 1 Department of Psychiatry and Medicine, University of Pittsburgh, Pittsburgh, Pennsylvania, USA; 2 Department of Population Health Sciences, Weill Cornell Medical College, New York, New York, USA; 3 Research Department, Crohn’s & Colitis Foundation, New York, New York, USA; 4 Health Economics and Outcomes Research Department, Optum, Eden Prairie, Minnesota, USA; 5 Department of Government Programs, Medical Director, Government Programs, Geisinger Health Plan, Danville, Pennsylvania, USA; 6 Department of Medicine, University of Michigan School of Medicine, Ann Arbor, Michigan, USA; 7 Institute for Healthcare Policy and Innovation, University of Michigan, Ann Arbor, Michigan, USA

**Keywords:** inflammatory bowel disease, Crohn’s disease, ulcerative colitis, chronic pain, opioids, narcotics, cost driver, healthcare resource utilization, healthcare costs

## Abstract

**Background:**

Opioid use by patients with inflammatory bowel disease (IBD) has been associated with poorer health outcomes. This study describes socioeconomic characteristics; health utilization trends; and costs of patients with IBD and either no opioid prescriptions, or in 1 of 3 opioid duration categories based on Center for Disease Control guidelines: acute (0–30 days), moderate (31–90 days), or chronic (>90 days). We utilized the Cost of IBD Care Optum research database results for this study.

**Methods:**

The Optum Research Database from years 2007 to 2016 including IBD patients with commercial or Medicare Advantage insurance in the United States was used. Additional inclusion criteria included continuous enrollment with medical and pharmacy benefit coverage for at least 24 months (12 months before and 12 months after the index date of IBD diagnosis). The association between costs and patient characteristics were assessed across a no opioid use group during this period and the 3 opioid duration groups.

**Results:**

Among 51,178 IBD patients, 33,229 (64.93%) were part of the no opioid use group, while 13,635 (26.64%) were in acute, 1698 (3.32%) were in moderate, and 2616 (5.11%) were in chronic use groups, as determined by pharmacy claims data. Patients in the chronic group were more likely to be white (75.38%) compared to all the other groups (no opioid use, acute, and moderate), have attained less education (only high school diploma), have had lower incomes, and have had Medicare instead of commercial insurance. Patients across all opioid prescription groups were more likely to have had diagnoses associated with pain in the prior year, with rates increasing by the length of opioid prescription (63.68%, 80.17%, and 86.11% for acute, moderate, and chronic groups). Compared to the no-use group, the acute group had more ambulatory (outpatient) visits, while the chronic group had fewer. Emergency department visits and inpatient hospitalizations were higher in all 3 opioid groups compared to the no opioid use group. Ambulatory, emergency department, inpatient, and total (medical + pharmacy) costs were higher in all 3 opioid groups, compared to the no opioid use group, even after adjusting for demographic and clinical patient characteristics.

**Conclusions:**

Among patients with IBD, increasing opioid use was associated with higher healthcare resource utilization and, concomitantly, higher healthcare costs during this period.

## INTRODUCTION

Patients with inflammatory bowel diseases (IBDs) have high rates of chronic abdominal pain,^1,2^ with rates as high as 57% in patients with Crohn’s disease (CD) and 33% in those with ulcerative colitis (UC).^3,4^ Patients with chronic pain syndromes, including IBD, are at significantly higher risk of chronic opioid use compared to community populations.^5–7^ Opioids have been prescribed to 5%–20% of IBD outpatients^8–11^ and up to 70% of IBD inpatients^12^; moreover, among hospitalized IBD patients, opioid use disorders were more common among those with CD, no or public insurance, and psychiatric comorbidities.^13^ In 1 study, 18% of younger patients with IBD (ages 15–29) received chronic opioid therapy^14^ and 5% of adults became heavy opioid users defined as a morphine equivalent dose of >50 mg/d within 10 years of initial IBD diagnosis.^6^

While there have been no prospective studies to date evaluating the prevalence of the DSM-V diagnosis of opioid use disorder in adults with IBD, in a retrospective analysis of a national insurance claims database, 35% of 15,119 patients with IBD developed persistent opioid use after an IBD flare requiring steroids.^15^ In patients followed over 1 year after an IBD admission, 4.7% were readmitted for an opioid use disorder 6% with CD and 2.6% with UC.^16^ In a retrospective study of 497 outpatients with UC seen over a 5-year period, 11.3% had chronic opioid use and 2.2% had opioid misuse, with the highest proportions in patient with concomitant functional gastrointestinal disorders.^17^

Besides chronic pain, female gender, comorbid history of psychiatric conditions, particularly depression and substance use disorders, 2 or more IBD-related surgeries, smoking, polypharmacy (being on ≥5 medications), abuse history (sexual, physical, or emotional), and functional disability have been identified as risk factors for chronic opioid use in this population.^6,9,18,19^

Multiple poor outcomes have been associated with opioid use in IBD. In a cross-sectional data study, IBD outpatients who used opioids had increased number of surgeries and hospital admissions, and reported lower quality of life compared to nonopioid users,^20^ and IBD patients prescribed opioids during an IBD-related hospitalization had no significant improvement in pain.^21^ Coates et al also found that opioid use was not associated with improved abdominal pain or quality of life in 542 IBD outpatients.^22^ In a 4-year registry study, IBD patients who had poor quality of life over the first 2 years were significantly more likely to be on opioids and have increased healthcare utilization the subsequent 2 years.^23^

Initiation of opioids to treat IBD-related pain can be associated with increased risk of continued opioid use in some patients. Dalal et al found a significant relationship between intravenous opioids during IBD hospitalizations and postdischarge opioid prescription with a dose-dependent effect.^24^ Pauly et al found that the strongest predictor for chronic opioid use among persons with newly diagnosed CD was previous opioid use^25^; emergency department (ED) utilization was also a predictor, and the authors noted that use of opioids can delay necessary treatment, which may lead to increased severity of disease and increased costs. Rates of opioid use disorders are rising in patients with IBD and are associated with increased length of hospitalizations (0.84 days medical and 2.79 days surgical).^13^ Opioid use has also been associated with increased risk of 30-day hospital readmission (odds ratio, 1.4) for IBD patients.^26^

Chronic opioid use has been associated with several negative outcomes in patients with IBD, including increased mortality, infection, postsurgical complications, and increased healthcare service utilization.^6,13,26–31^ Not surprisingly, opioid use, particularly chronic use, predicted high healthcare costs among IBD patients in several studies.^27,32,33^ While the literature to date has shown negative outcomes and increased medical utilization linked to opioid use in the IBD population, it has not centered on the related impact on healthcare costs. We sought to use nationally representative commercial and Medicare Advantage claims data to better understand the role that opioid use plays in driving up the already high healthcare costs associated with IBD,^34^ by conducting a more detailed analysis of the relationship between frequency of opioid use and healthcare costs, accounting for demographic, socioeconomic, and clinical characteristics. Describing the potential cost consequences of opioids can help provide motivation for providers, administrators, and payers to focus on and provide resources to this issue.

## METHODS

### Participants

The study was designed as a retrospective analysis using administrative claims data from the Optum Research Database for commercial and Medicare Advantage beneficiaries, who had an index date of first IBD diagnosis between January 2007 and December 2015, and were continuously enrolled with medical and pharmacy benefit coverage for a minimum of 12 months prior to and following their index date (see Ref. ^34^ for methodological details). Patients with metastatic cancer or HIV/AIDs (n = 1604) were excluded from the analyses.

Beneficiaries were stratified according to the number of opioid prescription days filled during each year following the index date, with the following thresholds: acute (1–30 days), moderate (31–90 days), and chronic (>90 days), based on ranges suggested by the Drug Enforcement Administration, Center for Disease Control (CDC), and prior research.^26^ Prescriptions that were Schedule IV or V Drug Enforcement Administration designations such as tramadol or low-dose codeine cough syrup formulations were not included due to low risk of misuse or dependence.^35,36^ Patients who fell into different opioid use classifications over time were included in the most chronic of those classifications. Persons with evidence of IBD, who had not filled a prescription for opioids during this period, were considered the no opioid use group. Annual utilization and cost outcomes were assessed based upon the beneficiary’s opioid use group in the corresponding year.

### Measures

Demographic information included age, sex, race, education level, and location of services. Socioeconomic measures included income (though 85% of cohort were missing this variable), health plan type (commercial vs Medicare Advantage, and plan structure), patient copay, and physician type. While data source information is not available, previous studies have used the same claims data approaches.^37^ Healthcare resource utilization included all-cause visits (ambulatory, office, ED, inpatient admissions, and other ancillary services), as well as utilization that resulted from IBD-specific claims. The site of service was determined from place of service codes and combined with revenue codes. Disease-specific utilization was calculated from ambulatory visits, ED visits, and inpatient admissions. Healthcare costs were operationalized as the sum of health plan and patient (deductible, copay, and coinsurance) expenditures. Costs categorized as pharmacy or medical. Medical costs included subcategories of ambulatory, ED, inpatient, and other. Pharmacy costs included the costs of prescription medications dispensed as recorded in pharmacy claims. Costs were adjusted to 2016 USD, using the annual medical care component of the Consumer Price Index. Data for the study included individual-level demographic and socioeconomic information linked to the claims data; specifically, race/ethnicity, education, household income, and household net worth. The data populating these socioeconomic elements are generated by a combination of self-report, modeling, census data, and a variety of other individual-level and population-level sources.

### Statistical Analyses

Comparisons between groups were assessed using chi-square tests for categorical variables and student independent *t* tests for continuous variables. Statistical significance was assessed at level of alpha = 0.05 with statistical corrections for multiple observations per patient. Since healthcare costs typically have a skewed distribution, a generalized linear model (with log link) was estimated on IBD-related costs to assess the effect of opioid use frequency, controlling for other patient characteristics (age, gender, sociodemographics, comorbid conditions, and treatments). The multivariable model (Supplementary Table 2) included in the adjustment covariates for IBD-related medications in the prior year, specifically 5-ASA, corticosteroid, immunosuppressant, biologic, antibiotics, nonsteroidal anti-inflammatory drugs, and steroid dependency. Most of the effects for IBD-related treatment showed some statistical significance, with the most notable that patients treated with biologics were associated with costs that were 3 times higher than patients without biologics.

## RESULTS

Table 1 provides demographic and clinical information by the 3 categories of opioid use. There were a total of 51,178 beneficiaries with a diagnosis of IBD; 33,229 (64.93%) did not fill an opioid prescription during the study period (“no opioid use group”). Beneficiaries who did fill an opioid prescription fell into the following categories, according to their longest period of prescribed use in at least 1 follow-up year: 13,635 (26.64%) filled for 30 days or less (“acute”); 1698 (3.32%) filled for 31–90 days (“moderate”); and 2616 (5.11%) filled for more than 90 days (“chronic”). Average age was slightly higher among chronic (53.27 years) and moderate (51.56 years) opioid use groups compared with the acute (47.61 years) and no opioid use (47.61 years) groups. The percent of patients who were female increased by level of opioid use, with the lowest being 51.90% female among patients in the no opioid use group and rising to a high of 66.70% in the chronic group. The majority of IBD beneficiaries were white (71.73%, n = 36,712), had at least a high school diploma (87.29%, n = 44,678), and were commercially insured (85.06%, n = 43,534). Over half (53.51%, n = 27,384) had a net worth of $150,000 or more. Beneficiaries in the chronic group were more likely to be white, compared to all the other groups (Table 1). Beneficiaries in all 3 opioid use groups were more likely to have a high school diploma alone, compared to the no opioid use group, but less likely than the no opioid use group to have a bachelor’s or an advanced degree. The no opioid use group was more likely to have a net worth over $25,000, including a net worth of at least $500,000. With regard to insurance type, no opioid use patients were significantly more likely to have a plan that included a health reimbursement (eg, flexible spending account) or health savings account, than any of the opioid groups.

**Table 1.** Patient Characteristics Within IBD Cohort—Patient Socioeconomic Status by Opioids

Demographics		Total (N = 51,178)	None (N = 33,229)	Acute: Opioids [1, 30] (N = 13,635)	Moderate: Opioids [31, 90] (N = 1698)	Chronic: Opioids >90 Days (N = 2616)	None vs Acute *P*	None vs Moderate *P*	None vs Chronic *P*
Age (continuous), years	n	51,178	33,229	13,635	1698	2616			
	Mean (SD)	48.03 (17.75)	47.61 (18.52)	47.61 (16.40)	51.56 (15.87)	53.27 (14.24)	0.976	<0.001	<0.001
Female	n	27,775	17,247	7739	1044	1745			
	%	54.27	51.90	56.76	61.48	66.70	<0.001	<0.001	<0.001
Region							<0.001	<0.001	<0.001
Northeast	n	7131	4713	1874	222	322			
	%	13.93	14.18	13.74	13.07	12.31	0.214	0.201	0.008
Midwest	n	14,754	9908	3726	433	687			
	%	28.83	29.82	27.33	25.50	26.26	<0.001	<0.001	<0.001
South	n	22,299	14,058	6242	811	1188			
	%	43.57	42.31	45.78	47.76	45.41	<0.001	<0.001	0.002
West	n	6994	4550	1793	232	419			
	%	13.67	13.69	13.15	13.66	16.02	0.119	0.972	<0.001
Race/ethnicity							<0.001	0.008	<0.001
White	n	36,712	23,757	9777	1206	1972			
	%	71.73	71.49	71.71	71.02	75.38	0.647	0.676	<0.001
African American/black	n	2502	1601	682	97	122			
	%	4.89	4.82	5.00	5.71	4.66	0.401	0.095	0.722
Hispanic	n	2402	1619	604	71	108			
	%	4.69	4.87	4.43	4.18	4.13	0.041	0.196	0.087
Asian	n	584	446	111	17	10			
	%	1.14	1.34	0.81	1.00	0.38	<0.001	0.231	<0.001
Other	n	610	469	119	10	12			
	%	1.19	1.41	0.87	0.59	0.46	<0.001	0.004	<0.001
Unknown	n	8368	5337	2342	297	392			
	%	16.35	16.06	17.18	17.49	14.98	0.003	0.118	0.148
Education							<0.001	<0.001	<0.001
<12th grade	n	331	212	87	14	18			
	%	0.65	0.64	0.64	0.82	0.69	0.999	0.350	0.757
High school diploma	n	13,190	8153	3562	557	918			
	%	25.77	24.54	26.12	32.80	35.09	<0.001	<0.001	<0.001
Some college or Associates degree	n	22,494	14,715	5936	705	1138			
	%	43.95	44.28	43.54	41.52	43.50	0.138	0.025	0.438
Bachelor’s/Graduate/Professional degree	n	8994	6239	2295	192	268			
	%	17.57	18.78	16.83	11.31	10.24	<0.001	<0.001	<0.001
Unknown	n	6169	3910	1755	230	274			
	%	12.05	11.77	12.87	13.55	10.47	<0.001	0.027	0.047
Income							<0.001	<0.001	<0.001
Under $40,000	n	1448	1065	237	42	104			
	%	2.83	3.21	1.74	2.47	3.98	<0.001	0.093	0.033
$40,000–49,999	n	635	461	127	14	33			
	%	1.24	1.39	0.93	0.82	1.26	<0.001	0.051	0.595
$50,000–59,999	n	663	498	113	23	29			
	%	1.30	1.50	0.83	1.35	1.11	<0.001	0.633	0.110
$60,000–74,999	n	954	720	169	16	49			
	%	1.86	2.17	1.24	0.94	1.87	<0.001	<0.001	0.318
$75,000–99,999	n	1374	1063	237	24	50			
	%	2.68	3.20	1.74	1.41	1.91	<0.001	<0.001	<0.001
$100,000+	n	3654	2845	647	48	114			
	%	7.14	8.56	4.75	2.83	4.36	<0.001	<0.001	<0.001
Unknown/missing	n	42,450	26,577	12,105	1531	2237			
	%	82.95	79.98	88.78	90.16	85.51	<0.001	<0.001	<0.001
Net worth (in $10,000s)							0.021	<0.001	<0.001
Under $25,000	n	5052	3100	1386	197	369			
	%	9.87	9.33	10.17	11.60	14.11	0.005	0.002	<0.001
$25,000–149,000	n	9356	5960	2519	333	544			
	%	18.28	17.94	18.47	19.61	20.80	0.169	0.080	<0.001
$150,000–249,000	n	6570	4253	1729	246	342			
	%	12.84	12.80	12.68	14.49	13.07	0.727	0.043	0.686
$250,000–499,000	n	11,279	7410	2979	361	529			
	%	22.04	22.30	21.85	21.26	20.22	0.285	0.315	0.014
$500,000+	n	9535	6405	2513	232	385			
	%	18.63	19.28	18.43	13.66	14.72	0.034	<0.001	<0.001
Unknown	n	9386	6101	2509	329	447			
	%	18.34	18.36	18.40	19.38	17.09	0.918	0.292	0.105
Insurance type							<0.001	<0.001	<0.001
Commercial	n	43,534	28,205	12,037	1376	1916			
	%	85.06	84.88	88.28	81.04	73.24	<0.001	<0.001	<0.001
Medicare Advantage	n	7644	5024	1598	322	700			
	%	14.94	15.12	11.72	18.96	26.76	<0.001	<0.001	<0.001
Plan type							<0.001	<0.001	<0.001
EPO	n	5496	3477	1594	173	252			
	%	10.74	10.46	11.69	10.19	9.63	<0.001	0.718	0.180
PPO	n	4767	2842	1377	206	342			
	%	9.31	8.55	10.10	12.13	13.07	<0.001	<0.001	<0.001
IND	n	1143	690	303	61	89			
	%	2.23	2.08	2.22	3.59	3.40	0.320	<0.001	<0.001
POS	n	27,599	18,495	7202	798	1104			
	%	53.93	55.66	52.82	47.00	42.20	<0.001	<0.001	<0.001
HMO	n	8914	5322	2653	351	588			
	%	17.42	16.02	19.46	20.67	22.48	<0.001	<0.001	<0.001
Other	n	3259	2403	506	109	241			
	%	6.37	7.23	3.71	6.42	9.21	<0.001	0.206	<0.001
Indicator for consumer-driven healthcare							<0.001	<0.001	<0.001
HRA (health reimbursement/flexible spending account)	n	2360	1626	598	56	80			
	%	4.61	4.89	4.39	3.30	3.06	0.019	0.003	<0.001
HSA (health savings account)	n	3120	2216	744	67	93			
	%	6.10	6.67	5.46	3.95	3.56	<0.001	<0.001	<0.001
No HRA/HSA	n	38,845	24,897	10,878	1288	1782			
	%	75.90	74.93	79.78	75.85	68.12	<0.001	0.389	<0.001
Missing	n	6853	4490	1415	287	661			
	%	13.39	13.51	10.38	16.90	25.27	<0.001	<0.001	<0.001

SD, standard deviation.

Patients in the opioid prescription groups were more likely to have had diagnoses associated with pain in the prior year, with rates increasing by the frequency of use (63.68%, 80.17%, and 86.11% for acute, moderate, and chronic groups); compared to 47.68% among no opioid use beneficiaries (Table 2). A higher percentage of the patients in each of the opioid prescription groups had baseline comorbidities associated with in-hospital mortality, as measured by the Quan-Charlson comorbidity score.^38^

**Table 2.** Clinical Characteristics by Opioids Cohort

Clinical Characteristics		Total (N = 51,178)	None (N = 33,229)	Acute: Opioids [1, 30] (N = 13,635)	Moderate: Opioids [31, 90] (N = 1698)	Chronic: Opioids >90 Days (N = 2616)	*P*
Baseline Charlson comorbidity score(continuous)	n	51,178	33,229	13,635	1698	2616	
	Mean (SD)	0.66 (1.23)	0.55 (1.13)	0.70 (1.24)	1.13 (1.52)	1.46 (1.77)	<0.001
Baseline Charlson comorbidity score (categorical)							<0.001
0	n	34,760	23,990	8896	815	1059	
	%	67.92	72.20	65.24	48.00	40.48	<0.001
1–2	n	12,304	7126	3582	630	966	
	%	24.04	21.45	26.27	37.10	36.93	<0.001
3–4	n	3071	1597	886	178	410	
	%	6.00	4.81	6.50	10.48	15.67	<0.001
5+	n	1043	516	271	75	181	
	%	2.04	1.55	1.99	4.42	6.92	<0.001
		Total person-years (N = 173,466)	None (N = 142,640)	Acute: Opioids [0, 30] (N = 21,719)	Moderate: Opioids [31, 90] (N = 3721)	Chronic: Opioids >90 days (N = 5386)	*P*
Prior year pain	n	89,505	68,011	13,831	2413	5250	
	%	51.60	47.68	63.68	80.17	86.11	<0.001

SD, standard deviation.

### Healthcare Resource Utilization and Costs

In terms of healthcare resource utilization (Table 3 and Supplementary Table 1), the no opioid use group, on average, had the fewest ambulatory, office, outpatient, and ED visits; inpatient stays and days; and unique medications. Concomitantly, the no opioid use group had the lowest average total healthcare costs among all groups, including IBD surgery-related costs. With the exception of ambulatory visits, which were highest among the acute group, the moderate group had the most visits. Consequently, the moderate group accrued the highest average total healthcare costs and was responsible for the largest out-of-pocket amount. This group also accrued the most IBD surgery-related costs. The no opioid use cohort had a higher rate of treatment over the follow-up with 5-ASA (aminosalicylate drugs), but a lower rate of treatment with corticosteroids or biologic medications than the opioid groups.

**Table 3.** Healthcare Resource Utilization Percent by Opioids: IBD-Related Claims at Follow-up Period, ALL IBD

		Total (N = 173,466)	None (N = 142,640)	Acute: Opioids [1, 30] (N = 21,719)	Moderate: Opioids [31, 90] (N = 3010)	Chronic: Opioids >90 Days (N = 6097)	None vs Acute *P*	None vs Moderate *P*	None vs Chronic *P*	Acute vs Moderate *P*	Acute vs Chronic *P*	Moderate vs Chronic *P*
Healthcare resource utilization												
Ambulatory visit	n	128,336	104,750	17,162	2263	4161						
	%	73.98	73.44	79.02	75.18	68.25	<0.001	0.053	<0.001	<0.001	<0.001	<0.001
Office visit	n	118,339	97,101	15,499	2044	3695						
	%	68.22	68.07	71.36	67.91	60.60	<0.001	0.862	<0.001	<0.001	<0.001	<0.001
Outpatient visit	n	72,381	57,062	11,302	1499	2518						
	%	41.73	40.00	52.04	49.80	41.30	<0.001	<0.001	0.145	0.030	<0.001	<0.001
Emergency room visit	n	16,220	10,606	3858	644	1112						
	%	9.35	7.44	17.76	21.40	18.24	<0.001	<0.001	<0.001	<0.001	0.494	0.001
Inpatient stay	n	19,532	11,314	5569	1007	1642						
	%	11.26	7.93	25.64	33.46	26.93	<0.001	<0.001	<0.001	<0.001	0.086	<0.001

The 3 opioid use groups had higher mean costs, compared to the no opioid use group, across the following categories: ambulatory, ED, and inpatient stays and days. Pharmacy costs were higher for persons in the acute and moderate groups, but not those in the chronic group, compared to the no opioid use group. Mean total costs (medical + pharmacy) were significantly greater among the acute ($19,871), moderate ($29,083), and chronic ($22,012) groups, compared to the no opioid use group ($9183). There was no consistent pattern of significantly different costs between the 3 opioid groups across different healthcare utilization categories (see Table 4 for details).

**Table 4.** Healthcare Costs by Opioids: IBD-Related Claims at Follow-up Period, ALL IBD (US $)

		Total (N = 173,466)	None (N = 142,640)	Acute: Opioids [1, 30] (N = 21,719)	Moderate: Opioids [31, 90] (N = 3010)	Chronic: Opioids >90 Days (N = 6097)	None vs Acute *P*	None vs Moderate *P*	None vs Chronic *P*	Acute vs Moderate *P*	Acute vs Chronic *P*	Moderate vs Chronic *P*
Healthcare costs ($)												
Medical costs	Mean (SD)	8182 (30,484)	6130 (25,591)	16,348 (40,193)	25,360 (59,562)	18,638 (54,979)	<0.001	<0.001	<0.001	<0.001	0.009	<0.001
	Median [IQR]	524 [68, 3707]	426 [53, 2851]	1979 [162, 15,652]	2497 [162, 24,101]	1062 [56, 13,763]						
Ambulatory costs	Mean (SD)	4007 (12,905)	3692 (12,314)	5585 (14,640)	5877 (16,973)	4844 (16,493)	<0.001	<0.001	<0.001	0.402	0.029	0.021
	Median [IQR]	322 [0, 2078]	292 [0, 1852]	723 [84, 3806]	567 [0, 4041]	311 [0, 2344]						
Office visit costs	Mean (SD)	1483 (6624)	1410 (6443)	1896 (7484)	2052 (8186)	1447 (6613)	<0.001	<0.001	0.794	0.387	0.002	0.004
	Median [IQR]	151 [0, 423]	146 [0, 400]	191 [0, 572]	199 [0, 625]	125 [0, 467]						
Outpatient costs	Mean (SD)	2524 (10,862)	2282 (10,333)	3689 (12,254)	3825 (14,289)	3397 (14,679)	<0.001	<0.001	<0.001	0.628	0.330	0.264
	Median [IQR]	0 [0, 1275]	0 [0, 1115]	78 [0, 2498]	0 [0, 2500]	0 [0, 1354]						
Emergency room costs	Mean (SD)	54 (456)	40 (372)	99 (559)	157 (974)	156 (1031)	<0.001	<0.001	<0.001	0.005	<0.001	0.981
	Median [IQR]	0 [0, 0]	0 [0, 0]	0 [0, 0]	0 [0, 0]	0 [0, 0]						
Inpatient stay costs	Mean (SD)	3738 (25,826)	2101 (21,207)	10,029 (34,751)	18,013 (52,130)	12,580 (49,291)	<0.001	<0.001	<0.001	<0.001	<0.001	<0.001
	Median [IQR]	0 [0, 0]	0 [0, 0]	0 [0, 2249]	0 [0, 12,596]	0 [0, 3469]						
Pharmacy costs	Mean (SD)	3135 (7168)	3053 (6880)	3523 (8226)	3723 (8401)	3374 (8809)	<0.001	<0.001	0.092	0.259	0.449	0.141
	Median [IQR]	509 [7, 3465]	508 [2, 3455]	650 [25, 3660]	565 [40, 3619]	209 [15, 2572]						
Total (medical + pharmacy) costs	Mean (SD)	11,317 (31,621)	9183 (26,705)	19,871 (41,640)	29,083 (60,530)	22,012 (56,047)	<0.001	<0.001	<0.001	<0.001	0.019	<0.001
	Median [IQR]	2896 [318, 9314]	2602 [274, 7916]	5331 [839, 22,410]	6124 [765, 32,142]	3791 [344, 21,424]						
Patient-paid costs	Mean (SD)	816 (1449)	760 (1347)	1096 (1734)	1217 (2544)	919 (1708)	<0.001	<0.001	<0.001	0.014	<0.001	<0.001
	Median [IQR]	312 [51, 918]	296 [45, 848]	445 [96, 1391]	422 [92, 1443]	271 [44, 1058]						
Plan-paid costs	Mean (SD)	10,501 (31,053)	8422 (26,194)	18,776 (40,931)	27,866 (59,657)	21,092 (55,253)	<0.001	<0.001	<0.001	<0.001	0.010	<0.001
	Median [IQR]	2314 [183, 8196]	2049 [151, 6902]	4561 [590, 20,386]	5397 [562, 30,226]	3278 [230, 19,890]						
Non-ER costs	Mean (SD)	11,263 (31,573)	9142 (26,677)	19,773 (41,570)	28,926 (60,428)	21,855 (55,907)	<0.001	<0.001	<0.001	<0.001	0.022	<0.001
	Median [IQR]	2862 [307, 9241]	2570 [267, 7873]	5269 [797, 22,232]	6073 [709, 31,859]	3640 [21,008]						
IBD surgery-related costs	Mean (SD)	1265 (12,096)	529 (7351)	4587 (21,195)	7024 (29,891)	3804 (27,978)	<0.001	<0.001	<0.001	<0.001	0.068	<0.001
	Median [IQR]	0 [0, 0]	0 [0, 0]	0 [0, 0]	0 [0, 0]	0 [0, 0]						

Costs are calculated among all patients, including patients with $0 cost in each Costs are adjusted using the annual medical care component of the Consumer Price Index (CPI) to reflect inflation to year 2016.

US Department of Labor, Bureau of Labor Statistics. Consumer Price Index. Medical Care. Series ID: CUUR0000SAM. Washington, DC: U.S. Dept. of Labor, Bureau of Labor Statistics, 2012. http://data.bls.gov/cgi-bin/surveymost?su.

IQR, interquartile range; SD, standard deviation.

Multivariable analyses (Supplementary Table 2) showed that after adjusting for other patient characteristics, costs were higher for patients in all opioid groups compared to patients in the no opioid group. Patients with acute use had costs 1.622 times higher than the no opioid group [95% confidence interval (CI): 1.588–1.658]; moderate use had 1.749 higher costs (95% CI: 1.651, 1.854); and chronic use had 1.603 higher costs (95% CI: 1.503–1.710).

## DISCUSSION

Several conclusions can be drawn from this analysis of claims data for a large cohort of adults with IBD. First, most IBD patients (65%) did not fill an opioid prescription during the 9-year study period. Persons without an opioid prescription, on average, used significantly fewer healthcare resources than persons who had filled an opioid prescription, and as a result, accrued the fewest costs. Overall, persons in the moderate group (1–3 months of opioid prescriptions) were the largest users of healthcare resources and accrued the most overall healthcare costs. Patients in the chronic opioid group had the highest rates of pain diagnoses in the year prior to opioid treatment and had the highest baseline comorbidity score. Differences in costs for opioid vs no opioid groups remained after adjusting for other patient characteristics, including the Quan-Charlson comorbidity score,^38^ although the effect size on costs appeared relatively similar across the opioid groups.

Pain is a known problem for many patients with IBD. In the Swiss IBD Cohort Study (n = 2152), 71% of patients reported pain during disease course, with 55% of patients with CD and 49% of patients with UC reporting pain for greater than 5 years.^2^ In this study, high proportions of the patients treated with moderate or chronic levels of opioids had diagnoses associated with pain in the prior year. However, as discussed above, from a clinical efficacy perspective, it is unclear that opioids are the solution for pain management for IBD. In the current study, beneficiaries differed across groups according to important demographic and socioeconomic characteristics, with lower socioeconomic status in opioid use groups compared to the nonopioid use category. Similarly, Click et al found that unemployment was significantly associated with higher healthcare costs, with the assumption being that socioeconomic disadvantage led to decreased longitudinal care for disease.^27^

Patients with IBD have been shown to be more likely than non-IBD patients to be prescribed an opioid in an acute care setting, such as an ambulatory care office or ED.^39^ In a prospective observational study of IBD outpatients followed over multiple years, opioid prescriptions significantly predicted future high healthcare utilization (odds ratio, 5.61).^27^ Given the changing legal regulations and climate around opioid prescribing since the time of data collection, prescribers have a need to increasingly focus on finding alternative methods of pain control for IBD patients, as well as to uncover active inflammation to respond to patients experiencing pain. The CDC guidelines suggest strongly that benefits outweigh the risks in considering initiation of opioids for noncancer chronic pain.^40^ However, it is important to acknowledge that there are individual patients with IBD who may benefit from appropriately prescribed opioids. Thus, in line with CDC guidelines, every effort should be made to keep patients on low dose and short duration opioids, if needed for acute conditions or postsurgery, and for chronic use, both reduction in pain severity and improvement in functioning should be monitored.^41^

### Strengths and Limitations

The primary strength of our study is the detailed and comprehensive nature of our data. There are several important limitations that must be considered as well. We acknowledge potential selection bias since Medicaid and uninsured patients were not included in the sample. Secondly, some IBD patients may have been misclassified in original claims data. There are always limitations in the use of claims data without corroborating data from the medical record. Third, socioeconomic measures included income but less than 15% of patients had his variable included. Fourth, opioid doses were not included in the analysis, such as ≥50 mg morphine equivalent—considered a high opioid dose, or any opioid dose plus a concurrent benzodiazepine prescription which also increases risk of poor outcomes.^42^ While we did not assess data evaluating the temporal relationship between IBD surgery and opioid use, the fact that the moderate opioid duration group had the highest IBD surgical costs, indicates it is highly unlikely all these additional costs are explained by appropriate acute postsurgical opioid use. It is possible that patients in the chronic opioid group were using small doses that were clinically helpful and thus not associated with higher costs. We could not account for opioid prescriptions outside of claims since some of these data were collected before mandated opioid monitoring began, and there is a possibility that some patients acquired opioids outside of a medical prescription. In addition, this study does not look at readmissions specifically, which is a topic of increasing interest in healthcare. In a recent study by Moreno et al of individuals with opioid use disorder, readmission rates at 20 and 90 days were found to be nearly 20% and 33%, respectively.^43^ Data does not include a measure of opioid use disorder. Additionally, the observation period for this study overlapped with drastic changes in state-level regulations and opioid prescribing practices, particularly since 2018, and in EDs and postsurgical settings. With the large sample size in the study, standard parametric tests were employed, since nonparametric tests are somewhat conservative in testing whether the distributions differ across groups; while healthcare costs typically have a skewed distribution, the large sample sizes allow the data to follow the central limit theorem and for the normal test distribution to apply. Similarly, consideration of the significance of the statistical tests should account for large sample sizes. The tests did not explicitly adjust for multiple test comparisons, but the majority of the statistically significant results were significant at *P* < 0.001, so that lowering the threshold for statistical significance would result in the same conclusions for most of the results.

We cannot conclude from these data whether there exists a causal relationship between opioid use and healthcare service utilization among IBD beneficiaries. The fact that patients with IBD who used opioids utilized services more frequently than nonopioid beneficiaries could indicate higher disease severity or a pattern of ED utilization stemming from a continuing need for pain reduction; however, the effect on cost remained after adjusting for comorbid illnesses. The value of this study is the examination of the impact of opioid use directly on healthcare costs. Future research evaluating why and by whom opioids were prescribed, and the appropriateness of the prescriptions is needed to implement data-driven strategies from a provider, system, and health plan payer perspective to develop narcotic mitigation approaches.

## CONCLUSIONS

In summary, overall opioid use of any duration was associated with significantly higher medical utilization and costs compared to no opioid use group without any opioid prescriptions. Every effort should be made to provide nonopioid pain medications and behavioral interventions, and, in instances where opioids are used, there should be ongoing screening for a use disorder.

## Supplementary Material

otab021_suppl_Supplementary_Material

## Data Availability

Data are not publicly available.

## Notes

Funding: Funding for this initiative is made possible through a grant from the Leona M. and Harry B. Helmsley Charitable Trust and UnitedHealth Group.

Conflict of Interest: Eva M. Szigethy has received funding as an educational speaker for Janssen Inc. Nicole M. Engel-Nitz—employee of Optum and shareholder in UnitedHealth Group. Has worked on unrelated studies in which funding was provided to Optum by GlaxoSmithKline, Genentech, AstraZeneca, Exact Sciences, Alexion, Janssen, Novartis, Eli Lilly and Company, EMD Serono, Bayer, Clovis, and Pfizer. Sean M. Murphy, Orna G. Ehrlich, Caren A. Heller, Perry Meadows, and John I. Allen: no conflicts.
