# Supplementary material for: Opioid Use Associated With Higher Costs Among Patients With Inflammatory Bowel Disease
Source: Crohns Colitis 360. 2021 Apr 14;3(2):otab021. doi: 10.1093/crocol/otab021 (PMC9802349; doi:10.1093/crocol/otab021)
Supplement: otab021_suppl_Supplementary_Material [file otab021_suppl_supplementary_material.docx]

**SUPPLEMENT**

**Supplement 1. Healthcare Resource Utilization Counts by Narcotic: IBD-related Claims at Follow-up Period, ALL IBD**

|  | | **Total (N=173,466)** | **None (N=142,640)** | **Acute: Opioids [1,30] (N=21,719)** | **Moderate: Opioids [31,90] (N=3,010)** | **Chronic: Opioids > 90 days (N=6,097)** | **None vs Acute p-value** | | **None vs Moderate p-value** | | **None vs Chronic p-value** | **Acute vs Moderate p-value** | | **Acute vs Chronic p-value** | | **Moderate vs Chronic p-value** |
| --- | --- | --- | --- | --- | --- | --- | --- | --- | --- | --- | --- | --- | --- | --- | --- | --- |
| **Healthcare resource counts** |  |  |  |  |  |  |  | |  | |  |  | |  | |  |
| Ambulatory visits | n | 173,466 | 142,640 | 21,719 | 3,010 | 6,097 |  | |  | |  |  | |  | |  |
|  | mean | 3.83 | 3.58 | 5.03 | 5.46 | 4.65 | <0.001 | | <0.001 | | <0.001 | 0.006 | | 0.011 | | <0.001 |
|  | SD | 5.11 | 4.68 | 6.33 | 7.29 | 7.21 |  | |  | |  |  | |  | |  |
| Office visits | n | 173,466 | 142,640 | 21,719 | 3,010 | 6,097 |  | |  | |  |  | |  | |  |
|  | mean | 2.41 | 2.27 | 3.03 | 3.35 | 2.98 | <0.001 | | <0.001 | | <0.001 | 0.002 | | 0.619 | | 0.004 |
|  | SD | 3.35 | 3.10 | 4.04 | 4.70 | 4.77 |  | |  | |  |  | |  | |  |
| Outpatient visits | n | 173,466 | 142,640 | 21,719 | 3,010 | 6,097 |  | |  | |  |  | |  | |  |
|  | mean | 1.44 | 1.33 | 2.03 | 2.13 | 1.69 | <0.001 | | <0.001 | | <0.001 | 0.214 | | <0.001 | | <0.001 |
|  | SD | 2.82 | 2.61 | 3.48 | 3.88 | 3.82 |  | |  | |  |  | |  | |  |
| Emergency room visits | n | 173,466 | 142,640 | 21,719 | 3,010 | 6,097 |  | |  | |  |  | |  | |  |
|  | mean | 0.13 | 0.09 | 0.27 | 0.39 | 0.37 | <0.001 | | <0.001 | | <0.001 | <0.001 | | <0.001 | | 0.464 |
|  | SD | 0.57 | 0.40 | 0.77 | 1.23 | 1.61 |  | |  | |  |  | |  | |  |
| Inpatient stays | n | 173,466 | 142,640 | 21,719 | 3,010 | 6,097 |  | |  | |  |  | |  | |  |
|  | mean | 0.16 | 0.10 | 0.38 | 0.62 | 0.48 | <0.001 | | <0.001 | | <0.001 | <0.001 | | <0.001 | | <0.001 |
|  | SD | 0.54 | 0.40 | 0.80 | 1.16 | 1.08 |  | |  | |  |  | |  | |  |
| Inpatient days | n | 173,466 | 142,640 | 21,719 | 3,010 | 6,097 |  | |  | |  |  | |  | |  |
|  | mean | 1.30 | 0.85 | 2.80 | 5.50 | 4.30 | <0.001 | | <0.001 | | <0.001 | <0.001 | | <0.001 | | <0.001 |
|  | SD | 7.51 | 6.56 | 8.58 | 14.75 | 14.04 |  | |  | |  |  | |  | |  |
| Pharmacy use, total number of unique medications | n | 173,466 | 142,640 | 21,719 | 3,010 | 6,097 |  | |  | |  |  | |  | |  |
|  | mean | 1.69 | 1.55 | 2.30 | 2.74 | 2.19 | <0.001 | | <0.001 | | <0.001 | <0.001 | | 0.001 | | <0.001 |
|  | SD | 1.51 | 1.42 | 1.70 | 1.87 | 1.75 |  | |  | |  |  | |  | |  |
| **Medication classes** |  |  |  |  |  |  |  |  | |  | | |  | |  |  |
| 5-ASA (aminosalicylate) | n | 83,297 | 70,027 | 10,145 | 1,250 | 1,875 |  |  | |  | | |  | |  |  |
|  | % | 48.02 | 49.09 | 46.71 | 41.53 | 30.75 | <0.001 | <0.001 | | <0.001 | | | <0.001 | | <0.001 | <0.001 |
| Corticosteroid | n | 74,600 | 55,527 | 12,965 | 2,115 | 3,993 |  |  | |  | | |  | |  |  |
|  | % | 43.01 | 38.93 | 59.69 | 70.27 | 65.49 | <0.001 | <0.001 | | <0.001 | | | <0.001 | | <0.001 | <0.001 |
| Immunosuppressant | n | 27,311 | 22,005 | 3,895 | 544 | 867 |  |  | |  | | |  | |  |  |
|  | % | 15.74 | 15.43 | 17.93 | 18.07 | 14.22 | <0.001 | <0.001 | | 0.114 | | | 0.870 | | <0.001 | <0.001 |
| Biologic | n | 20,931 | 15,947 | 3,504 | 542 | 938 |  |  | |  | | |  | |  |  |
|  | % | 12.07 | 11.18 | 16.13 | 18.01 | 15.38 | <0.001 | <0.001 | | <0.001 | | | 0.020 | | 0.387 | 0.015 |

**Supplement 2. Generalized Linear Model Results on IBD-Related Costs**

| **Independent Variables** | **Dependent Variable (Total Costs, All Cause)** | | | |
| --- | --- | --- | --- | --- |
|  | **cost ratio** | **lower 95% CI** | **upper 95% CI** | **p-value** |
| Cohort UC or CD | 0.972 | 0.948 | 0.996 | 0.022 |
| **Calendar year** |  |  |  | <0.001 |
| 2007 | – | – | – | – |
| 2008 | 0.896 | 0.871 | 0.922 | <0.001 |
| 2009 | 0.921 | 0.891 | 0.952 | <0.001 |
| 2010 | 0.908 | 0.882 | 0.934 | <0.001 |
| 2011 | 0.940 | 0.911 | 0.970 | <0.001 |
| 2012 | 0.924 | 0.897 | 0.953 | <0.001 |
| 2013 | 0.927 | 0.897 | 0.957 | <0.001 |
| 2014 | 0.964 | 0.930 | 0.998 | 0.039 |
| 2015 | 0.983 | 0.944 | 1.023 | 0.389 |
| 2016 | 0.977 | 0.820 | 1.164 | 0.797 |
| **Age** |  |  |  | <0.001 |
| <5 | 1.534 | 1.209 | 1.946 | <0.001 |
| 6-12 | 1.531 | 1.405 | 1.669 | <0.001 |
| 13-18 | 1.426 | 1.312 | 1.551 | <0.001 |
| 19-24 | 1.124 | 1.072 | 1.178 | <0.001 |
| 25-34 | 1.017 | 0.981 | 1.055 | 0.364 |
| 35-44 | – | – | – | – |
| 45-54 | 1.048 | 1.014 | 1.084 | 0.006 |
| 55-64 | 1.173 | 1.120 | 1.229 | <0.001 |
| 65-74 | 1.397 | 1.225 | 1.594 | <0.001 |
| 75+ | 1.565 | 1.405 | 1.742 | <0.001 |
| **Gender** |  |  |  | <0.001 |
| Female | 0.901 | 0.876 | 0.925 | <0.001 |
| Male | – | – | – | – |
| **Region** |  |  |  |  |
| Northeast | – | – | – | – |
| Midwest | 0.922 | 0.888 | 0.958 | <0.001 |
| South | 0.837 | 0.809 | 0.866 | <0.001 |
| West | 0.929 | 0.881 | 0.981 | 0.008 |
| Other | 1.000 | 1.000 | 1.000 | – |
| **Urban/Rural** |  |  |  | 0.003 |
| Urban | – | – | – | – |
| Rural | 0.974 | 0.920 | 1.031 | 0.366 |
| Missing | 1.159 | 1.039 | 1.294 | 0.008 |
| **Race** |  |  |  | 0.615 |
| White | – | – | – | – |
| African-American/Black | 1.058 | 0.988 | 1.133 | 0.107 |
| Hispanic | 1.013 | 0.957 | 1.073 | 0.646 |
| Asian | 1.010 | 0.892 | 1.144 | 0.876 |
| Other | 0.973 | 0.900 | 1.052 | 0.488 |
| Unknown | 0.994 | 0.956 | 1.032 | 0.738 |
| **Education level** |  |  |  | 0.197 |
| <12th grade | – | – | – | – |
| High School diploma | 0.958 | 0.804 | 1.141 | 0.629 |
| Some college or Associates degree | 0.977 | 0.820 | 1.165 | 0.799 |
| Bachelor's/Graduate/Professional degree | 1.022 | 0.848 | 1.232 | 0.818 |
| Unknown | 0.938 | 0.783 | 1.124 | 0.490 |
| **Net worth** |  |  |  | 0.054 |
| Under $25,000 | – | – | – | – |
| $25,000-149,000 | 1.042 | 0.994 | 1.092 | 0.084 |
| $150,000-249,000 | 1.001 | 0.958 | 1.046 | 0.971 |
| $250,000-499,000 | 1.007 | 0.962 | 1.054 | 0.771 |
| $500,000+ | 1.031 | 0.963 | 1.102 | 0.380 |
| Unknown | 1.059 | 1.008 | 1.113 | 0.024 |
| **Plan type** |  |  |  | <0.001 |
| EPO | 1.002 | 0.972 | 1.034 | 0.886 |
| PPO | 0.998 | 0.955 | 1.044 | 0.945 |
| IND | 1.109 | 0.970 | 1.268 | 0.129 |
| POS | – | – | – | – |
| HMO | 0.937 | 0.908 | 0.967 | <0.001 |
| Other | 0.387 | 0.314 | 0.476 | <0.001 |
| Medicare | 0.543 | 0.472 | 0.624 | <0.001 |
| **Indicator for consumer driven healthcare** |  |  |  |  |
| H.R.A | 0.899 | 0.869 | 0.930 | <0.001 |
| H.S.A | 0.941 | 0.902 | 0.981 | 0.004 |
| NO H.R.A / H.S.A | – | – | – | – |
| CDHP missing | 1.000 | 1.000 | 1.000 | – |
| **HCRU** |  |  |  | <0.001 |
| Previous year Charlson score | 1.031 | 1.009 | 1.054 | 0.006 |
| Previous year ER visit | 1.062 | 1.040 | 1.085 | <0.001 |
| Previous year Inpatient visit | 0.977 | 0.945 | 1.011 | 0.177 |
| Previous year Total cost related diseases (Highest 25%) | 1.161 | 1.130 | 1.193 | <0.001 |
| Previous year Relapse | 0.921 | 0.888 | 0.955 | <0.001 |
| **Previous year Provider specialty** |  |  |  | <0.001 |
| Previous year Gastroenterologist | 0.927 | 0.909 | 0.946 | <0.001 |
| Previous year Immunologist | 1.024 | 0.978 | 1.072 | 0.316 |
| Previous year Mental healthcare | 1.051 | 1.015 | 1.087 | 0.005 |
| **Previous year Specific comorbidities** |  |  |  |  |
| Previous year Pregnancy (if female) | 1.170 | 1.115 | 1.228 | <0.001 |
| Previous year Myocardial infarction | 1.032 | 0.933 | 1.143 | 0.537 |
| Previous year Congestive heart failure | 1.046 | 0.950 | 1.152 | 0.358 |
| Previous year Peripheral vascular disease | 1.063 | 1.023 | 1.105 | 0.002 |
| Previous year Cerebrovascular disease | 1.016 | 0.955 | 1.080 | 0.618 |
| Previous year Dementia | 0.966 | 0.904 | 1.031 | 0.299 |
| Previous year Chronic pulmonary disease | 1.016 | 0.977 | 1.056 | 0.429 |
| Previous year Rheumatic disease | 1.062 | 1.021 | 1.106 | 0.003 |
| Previous year Peptic ulcer disease | 1.061 | 0.985 | 1.142 | 0.119 |
| Previous year Mild liver disease | 1.031 | 0.980 | 1.084 | 0.241 |
| Previous year Diabetes (with and without chronic complications) | 1.067 | 1.006 | 1.133 | 0.032 |
| Previous year Hemiplegia or paraplegia | 1.451 | 0.813 | 2.591 | 0.208 |
| Previous year Renal disease | 1.188 | 1.115 | 1.266 | <0.001 |
| Previous year Any malignancy including lymphoma and leukemia, except malignant neoplasm of the skin | 1.187 | 1.098 | 1.283 | <0.001 |
| Previous year Moderate or severe liver disease | 1.204 | 0.927 | 1.563 | 0.165 |
| Previous year AIDS/HIV | 1.000 | 1.000 | 1.000 | – |
| Previous year Psychiatric disorders (depression, anxiety, etc.) | 0.999 | 0.976 | 1.023 | 0.951 |
| Previous year Anemia | 1.087 | 1.054 | 1.121 | <0.001 |
| Previous year Hypertension | 0.971 | 0.943 | 1.000 | 0.053 |
| Previous year Asthma | 0.957 | 0.894 | 1.023 | 0.196 |
| Previous year Gastroesophageal reflux disease (GERD) | 0.968 | 0.946 | 0.991 | 0.007 |
| Previous year Headache | 1.010 | 0.965 | 1.057 | 0.676 |
| Previous year Osteoarthritis | 1.020 | 0.991 | 1.050 | 0.181 |
| Previous year Urinary tract infections (UTI) | 1.011 | 0.976 | 1.047 | 0.551 |
| Previous year Pain | 1.061 | 1.041 | 1.081 | <0.001 |
| Previous year Extraintestinal manifestations | 0.963 | 0.912 | 1.016 | 0.169 |
| **Previous year AHRQ Comorbidities** |  |  |  | <0.001 |
| Previous year Disorders of lipid metabolism | 0.957 | 0.931 | 0.984 | 0.002 |
| Previous year Respiratory infections | 0.968 | 0.951 | 0.985 | <0.001 |
| Previous year Other connective tissue disease | 1.018 | 1.000 | 1.036 | 0.050 |
| Previous year Eye disorders | 1.021 | 1.001 | 1.042 | 0.040 |
| Previous year Spondylosis; intervertebral disc disorders; other back problems | 1.001 | 0.980 | 1.023 | 0.927 |
| Previous year Other lower respitory disease | 1.032 | 1.008 | 1.057 | 0.008 |
| Previous year Diseases of the female genital organs | 1.016 | 0.997 | 1.035 | 0.101 |
| Previous year Other nutritional; endocrine; and metabolic disorders | 1.051 | 1.023 | 1.080 | <0.001 |
| Previous year Ear conditions | 1.012 | 0.986 | 1.039 | 0.362 |
| Previous year Other upper respiratory disease | 0.980 | 0.952 | 1.008 | 0.163 |
| Previous year Other nervous system disorders | 1.039 | 1.012 | 1.066 | 0.004 |
| Previous year Thyroid disorders | 0.991 | 0.965 | 1.019 | 0.531 |
| **Medication classes** |  |  |  | <0.001 |
| Number of unique medications | 1.070 | 1.067 | 1.074 | <0.001 |
| 5-ASA | 1.080 | 1.059 | 1.101 | <0.001 |
| Corticosteroid | 1.196 | 1.176 | 1.216 | <0.001 |
| immunosuppressant | 1.018 | 0.995 | 1.042 | 0.121 |
| Biologic | 3.085 | 3.003 | 3.170 | <0.001 |
| Antibiotics | 1.152 | 1.131 | 1.173 | <0.001 |
| NSAIDs | 0.924 | 0.907 | 0.942 | <0.001 |
| Steroid dependency | 1.155 | 1.126 | 1.185 | <0.001 |
| **Narcotic** |  |  |  | <0.001 |
| None | – | – | – | – |
| Acute: Opioid [0,30] | 1.622 | 1.588 | 1.658 | <0.001 |
| Moderate: Opioid [31,90] | 1.749 | 1.651 | 1.854 | <0.001 |
| Chronic: Opioid > 90 days | 1.603 | 1.503 | 1.710 | <0.001 |
| Observations read = 173,466, Observations used = 173,466 Pearson chi-square=511051.337, DF=173362 Specification link test: p-value=<0.001 Park test: estimate = 1.609, gamma distribution p-value = <0.001 Park test p-value for Normal distribution: <0.001 Park test p-value for Poisson distribution: <0.001 Park test p-value for Gamma distribution: <0.001 Park test p-value for Wald or Inverse Gaussian distribution: <0.001 | | | | |
